# Integrative Analysis of DNA Methylation and Transcriptome Identifies a Predictive Epigenetic Signature Associated With Immune Infiltration in Gliomas

**DOI:** 10.3389/fcell.2021.670854

**Published:** 2021-05-31

**Authors:** Jianlei Zhang, Jiang Yin, Liyun Luo, Danqing Huang, Dongfeng Zhai, Ge Wang, Ning Xu, Mingqiang Yang, Ying Song, Guopei Zheng, Qiong Zhang

**Affiliations:** Affiliated Cancer Hospital & Institute of Guangzhou Medical University, Guangzhou Municipal and Guangdong Provincial Key Laboratory of Protein Modification and Degradation, Guangzhou Key Laboratory of “Translational Medicine on Malignant Tumor Treatment”, Guangzhou, China

**Keywords:** glioma, epigenetic signature, prognosis, immune infiltration, multi-omics integration

## Abstract

Glioma is the most common primary brain tumor with poor prognosis and high mortality. The purpose of this study was to use the epigenetic signature to predict prognosis and evaluate the degree of immune infiltration in gliomas. We integrated gene expression profiles and DNA methylation data of lower-grade glioma and glioblastoma to explore epigenetic differences and associated differences in biological function. Cox regression and lasso analysis were used to develop an epigenetic signature based on eight DNA methylation sites to predict prognosis of glioma patients. Kaplan–Meier analysis showed that the overall survival time of high- and low-risk groups was significantly separated, and ROC analysis verified that the model had great predictive ability. In addition, we constructed a nomogram based on age, sex, 1p/19q status, glioma type, and risk score. The epigenetic signature was obviously associated with tumor purity, immune checkpoints, and tumor-immune infiltrating cells (CD8+ T cells, gamma delta T cells, M0 macrophages, M1 macrophages, M2 macrophages, activated NK cells, monocytes, and activated mast cells) and thus, it may find application as a guide for the evaluation of immune infiltration or in treatment decisions in immunotherapy.

## Introduction

Gliomas are the most common primary tumors of the central nervous system, accounting for 60% of craniocerebral tumors (Ostrom et al., 2019). According to the malignant features, the World Health Organization (WHO) divides gliomas into four grades. With the continuous discovery of new molecular markers, the accurate classification of gliomas has been promoted. Currently, the WHO recommends that molecular markers such as co-deletion of chromosome arms 1p and 19q (1p/19q co-deletion) and isocitrate dehydrogenase (IDH) mutation status be included in the histopathological classification of gliomas (Louis et al., 2016). Surgery combined with chemotherapy or radiotherapy is a common treatment strategy for gliomas, but the prognosis varies greatly (van den Bent et al., 2013; van den Bent, 2014; Buckner et al., 2016). In particular, glioblastoma (GBM), or grade 4 glioma, is the most common and fatal malignant brain tumor, with a short median survival of only 12 to 15 months (Hanif et al., 2017). Therefore, the development of effective biomarkers for risk assessment may help to guide the choice of future targeted treatment strategies.

The complex tumor microenvironment (TME) has a serious influence on the gene expression in cancer tissue, affecting disease progression and clinical outcome (Cooper et al., 2012; Yoshihara et al., 2013). In the progression of gliomas, due to the destruction of the blood-brain barrier, tumor cells secrete a large number of chemokines that recruit immune cells from the peripheral blood, and the proportion of microglia/macrophages in the tumor cavity increases, which is associated with the malignant degree of gliomas (Yi et al., 2011). Therefore, the evaluation of immune infiltration of gliomas plays an important role in monitoring the progression of gliomas, and tumor-infiltrating immune cells have the potential to be used as drug targets to improve the prognosis of glioma patients (Xiong et al., 2018). Immunotherapy suppresses the rejection of tumor cells by the host immune system by inducing, enhancing, or suppressing the immune response, and thus uses the host immune system to kill tumor cells. At present, immune checkpoint inhibitors, such as anti-CTLA-4 monoclonal antibody (ipilimumab) and anti-PD-1 monoclonal antibody (nivolumab), have achieved good results in tumor therapy (O’Day et al., 2010; Rizvi et al., 2015). However, research on immune checkpoint inhibitors in glioma is still insufficient, and new drugs are currently being evaluated in animal models and in clinical trials (Omuro et al., 2018; Crommentuijn et al., 2020; Ladomersky et al., 2020).

DNA methylation plays an important role in maintaining the structural stability of the genome and in regulating gene expression. Epigenetic modification of DNA, including abnormal DNA methylation, especially in the promoter region of genes as such as CDKN2A, has been reported to be associated with tumorigenesis and has great potential as a tumor biomarkers (Malzkorn et al., 2011; Klutstein et al., 2016; Pan et al., 2018) with numerous advantages. First, epigenetic information is more stable than RNA and protein, and is not vulnerable to physical and chemical damage (Issa, 2012). Second, abnormally altered DNA methylation is an early event of carcinogenesis that predates genetic defects and abnormal gene expression. Detection of DNA methylation changes may provide a more timely and accurate assessment of cancer progression (Fleischer et al., 2014; Timp et al., 2014). Third, the development of drugs to reverse epigenetic modification has great potential for cancer treatment (Zhu and Yao, 2009). The large number of samples and data regarding genome-wide methylation sites stored in The Cancer Genome Atlas (TCGA) database provides a source for the identification of identify biomarkers.

Multi-omics integration analysis interrogates previously isolated data relative to degree of DNA methylation and RNA expression, which may provide new insights into the pathogenesis and treatment of gliomas (Ceccarelli et al., 2016; Binder et al., 2019). We explored epigenetic differences between lower-grade glioma (LGG) and glioblastomas (GBM) based on integrated expression profile data and DNA methylation data in the promoter region. Using a series of statistical methods, we constructed a novel epigenetic signature based on eight DNA methylation sites. Furthermore, we evaluated the ability of the signature to predict survival outcomes and its association with immune infiltration in gliomas. Finally, to apply the epigenetic signature to clinical practice, we integrated a series of independent prognostic factors to construct a nomogram.

## Materials and Methods

### Datasets Extracted From TCGA and GEO Databases

The DNA methylation data, gene expression data, and corresponding clinical information of glioma patients (including LGG and GBM samples) were obtained from the TCGA database^1^. In addition, we downloaded the LGG dataset (GSE104293) and the GBM dataset (GSE48462) from the GEO database^2^. DNA methylation data were generated using the Infinium Human Methylation 450 BeadChip covering 485,577 DNA methylation sites. For each CpG site, the β value represented the DNA methylation level from 0 (no methylation) to 1 (100% methylation). CpGs in the promoter regions located 2 kb upstream to 0.5 kb downstream from transcription start sites (TSS) covering 145,907 DNA methylation sites were selected for the present study.

### Identification of Genes, and DNA Methylation Sites, and the Epigenetic Genes Involved in Glioma Progression

Using fold change >2 or <0.5 and a false discovery rate (FDR) <0.01 as the threshold, gliomas samples were analyzed to identify differentially expressed genes between LGG samples and GBM samples using the edgeR package^3^. Based on the thresholds β value >0.2 and FDR <0.01, different DNA methylation sites were identified using the Limma package^4^ (Ritchie et al., 2015). The correlation between differentially upregulated genes and the degree of reduced methylation on sites on promotors of downregulated genes, and conversely, the downregulate differentially expressed genes and the degree of methylation of promotors sites was analyzed according to the standard of correlation <−0.3 and FDR <0.01. Finally, we identified epigenetic genes associated with glioma progression including epigenetically-induced genes (low promoter methylation with high gene expression) and epigenetically-suppressed genes (high promoter methylation with low gene expression).

### Functional Enrichment Analysis of Epigenetic Genes

To explore the biological implications of epigenetic genes in glioma progression, functional enrichment analyses including gene ontology (GO) hierarchy and Kyoto Encyclopedia of Gene and Genomes (KEGG) pathways were performed using the Database for Annotation, Visualization, and Integrated Discovery^5^ using *P* < 0.05 as a significance threshold.

### Construction of a Prognosis Risk Signature Based on DNA Methylation Sites of Epigenetically-Regulated Genes

Glioma samples were randomly assigned to training and validation data sets using the caret package^6^. A training data set was used to develop a risk model, and a validation data set and a LGG dataset (GSE104293) and GBM dataset (GSE48462) were used to verify the model. Univariate‘ cox regression was used to screen for DNA methylation sites that had a significant effect on prognosis, and the filter condition was set at *P* < 0.05. Lasso regression can reduce the complexity of the model by adjusting the parameters of the model to avoid over-fitting by the glmnet package^7^ (Simon et al., 2011). The degree of complexity adjustment of LASSO regression was controlled by the parameter λ–the greater the λ, the greater the punishment for the linear model with more variables—to obtain a model with fewer variables. Finally, we used multivariate Cox regression analysis to screen for independent prognostic factors, which could be used to develop a risk prognostic model. A risk score formula was defined by the sum of the products of the β value of each DNA methylation site and the correlation coefficient. Based on the median value of risk, patients are divided into two groups: high- and low-risk groups. Finally, we evaluated the prediction performance of the signature in the internal and external validation datasets (LGG: GSE104293 and GBM: GSE48462).

### Gene Set Enrichment Analysis

Gene set enrichment analysis (GSEA) was preformed to analyze differences between high- and low-risk groups using the “c2 cp keg v7.0 symbols collection” as the reference gene set (Subramanian et al., 2005). A FDR <0.05 after performing 1,000 permutations was considered to significantly enriched.

### ESTIMATE Algorithm

The ESTIMATE algorithm was applied to analyze the expression characteristics of specific genes in immune cells and stromal cells, and to calculate immune and stromal scores to evaluate the degree of invasion of non-tumor cells and tumor purity, and to compare differences between high- and low-risk groups (Bindea et al., 2013).

### CIBERSORT Algorithm

CIBERSORT, a deconvolution algorithm, evaluates the gene composition of each immune cell by calculating the expression level of each gene in each immune cell. In other words, the adjusted expression profiles of complex tissues were used to predict the proportion of 22 tumor immune infiltrating cell (TIICs) types (Newman et al., 2015). The r package of CIBERSORT was used to calculate each the proportion of TIIC types in each sample, and non-conforming samples were filtered out when the *P*-value was <0.05. Using the filtered data, the types of TIIL cells were compared in the high- and low-risk groups.

### Construction and Assessment of the Nomogram

To apply the model to the clinic, we integrated a series of indicators to construct a nomogram. We used univariate Cox regression to analyze a series of indicators (age, sex, *IDH* mutation status, *ATRX* mutation status, 1p/19q co-deletion status, promoter methylation status of *MGMT*, radiation therapy history, and glioma subtype) to screen for prognostic factors associated with the overall survival (OS) of patients with glioma, and then multivariate Cox regression was performed to screen for independent prognostic factors. Finally, a nomogram was constructed to predict the 1-, 3-, and 5-year OS rates of glioma patients using the rms package^8^. We constructed receiver operating characteristic (ROC) curves to assess the 1-, 3-, and 5-year prediction performance of the nomogram.

### Immunohistochemistry

Glioma paraffin-embedded tissues were collected from the Affiliated Cancer Hospital of Guangzhou Medical University (LGG, *n* = 16; high-grade gliomas, *n* = 17). Immunohistochemical staining of the target proteins was performed on paraffin-embedded tissues using an anti-SPON2 antibody (Proteintech, #20513-1-AP), anti-IFI44 antibody (Proteintech, #7233-1-AP), anti-CD68 antibody (Abcam, #ab125212), anti-CD206 antibody (CST, #91992S). Then, the slices were stained with diaminobenzidine (DAB) and counterstained with hematoxylin. The scoring of tumor cells was as follows: negative (0), yellowish (1–4), light brown (5–8), and dark brown (9–12). The differences in expression of four genes in low-grade and high-grade gliomas were compared by unpaired Student’s t-test, and their association was evaluated by Pearson’s correlation analysis. This study was approved by the Ethics Committee of the Affiliated Cancer Hospital of Guangzhou Medical University. Informed consent was obtained from each patient.

### Statistical Analysis

All statistical analyses were carried out using R software. The Chi-square test was used to analyze the association between the epigenetic signature and common clinical features. The prediction accuracy of the signature was verified by Kaplan–Meier (K-M) analysis and ROC curve analysis. *P* < 0.05 was considered statistically significant. The area under the curve (AUC) value was used as the evaluation standard of accuracy.

## Results

### Data Collection and Difference Analysis

**FIGURE 1:**
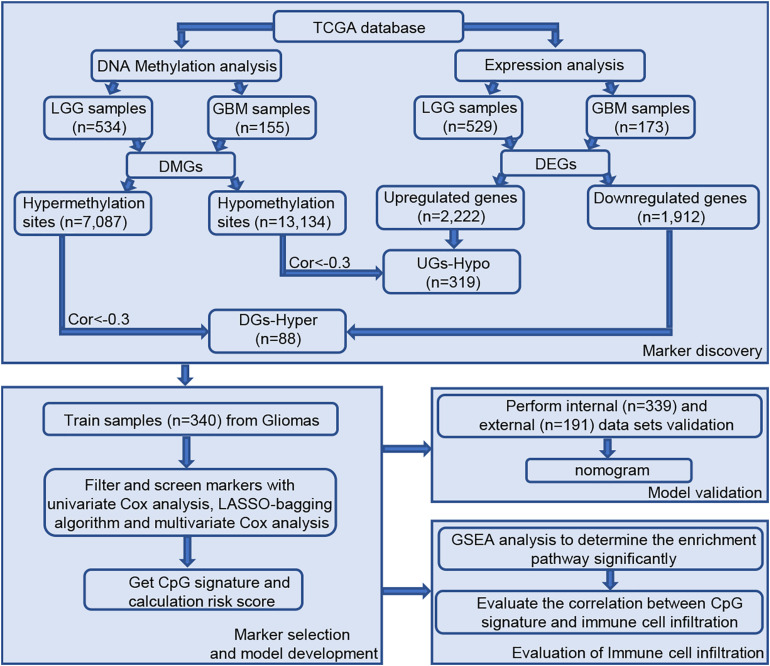
Workflow of study design. Abbreviations: TCGA, The Cancer Genome Atlas; EI, epigenetically-induced genes; ES, epigenetically suppressed genes.

A total of 689 glioma samples, consisting of 534 LGG and 155 GBM samples, with information on 485,577 DNA methylation sites was obtained from TCGA. Because DNA methylation in promoter regions significantly affects gene expression, we analyzed the TSS200 and TSS1500 sites located in transcription start sites, and as a result, 145,907 methylation sites were filtered for subsequent study. As shown in the workflow diagram (Figure 1), compared with the LGG samples, 20,220 differential DNA methylation sites were screened from GBM samples (7087 upregulated DNA methylation sites and 13,134 downregulated DNA methylation sites). In addition, we analyzed differentially expressed genes in glioma patients, including 529 LGG samples and 173 GBM samples. A total of 4,134 different expression genes were obtained between LGG samples and GBM samples based on above screening criteria (2,222 upregulated genes and 1,912 downregulated genes).

### Identification of Epigenetic Genes Using Correlation Analysis

The degree of gene expression is regulated by DNA methylation. Hypermethylation inhibits the expression of downstream genes, while hypomethylation promotes the expression of downstream genes. Overall, 592 samples with both expression profile and methylation data were selected for follow-up correlation analysis. First, we considered the intersection of highly methylation genes and downregulated genes, and the intersection of low methylation genes and upregulated genes. Next, using the standard of correlation <−0.3 and FDR < 0.01, a group of 407 epigenetic genes was identified, including 319 epigenetically-induced genes (low promoter methylation with high gene expression) and 88 epigenetically-suppressed genes (high promoter methylation with low gene expression).

### GO and Pathway Enrichment Analysis of Epigenetic Genes

**FIGURE 2:**
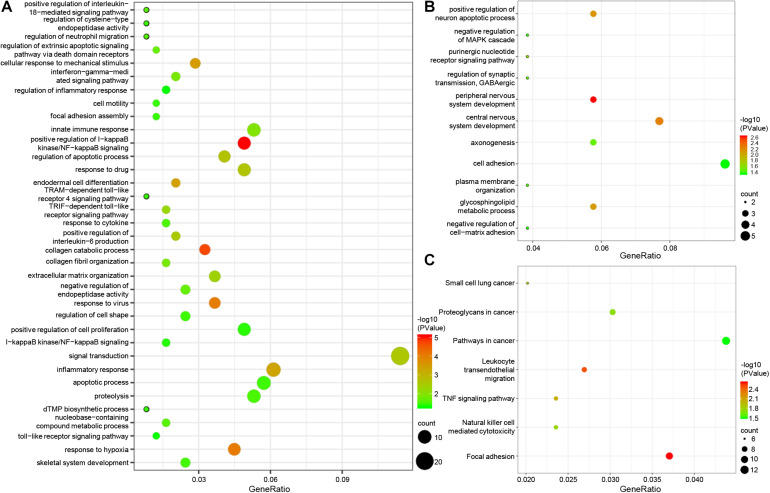
GO and pathway enrichment analysis of epigenetic genes. **(A)** biological process of epigenetically induced genes. **(B)** Biological processes of epigenetically suppressed genes. **(C)** KEGG pathway analysis of epigenetic genes.

To further explore the function of the screened epigenetic genes, the online software DAVID was used for GO analysis of epigenetic genes. GO analysis results classified epigenetic genes functions into three functional groups: biological processes, cellular components, and molecular function. We focused on the biological processes of epigenetically-regulated genes. The results showed that epigenetically-induced genes were enriched in ‘positive regulation of I-kappaB kinase/NF-kappaB signaling’, ‘regulation of apoptotic process’, ‘innate immune response’, and ‘inflammatory response’ (Figure 2A), while epigenetically suppressed genes were enriched in ‘peripheral nervous system development’, ‘central nervous system development’, and ‘positive regulation of neuron apoptotic process’ (Figure 2B). KEGG pathway analysis defined the most significantly enriched pathways of the epigenetically-regulated genes, were found to be enriched in ‘TNF signaling pathway’, ‘Proteoglycans in cancer,’ and ‘Pathways in cancer’ (Figure 2C).

### Establishment of the Epigenetic Signature and Validation

**FIGURE 3:**
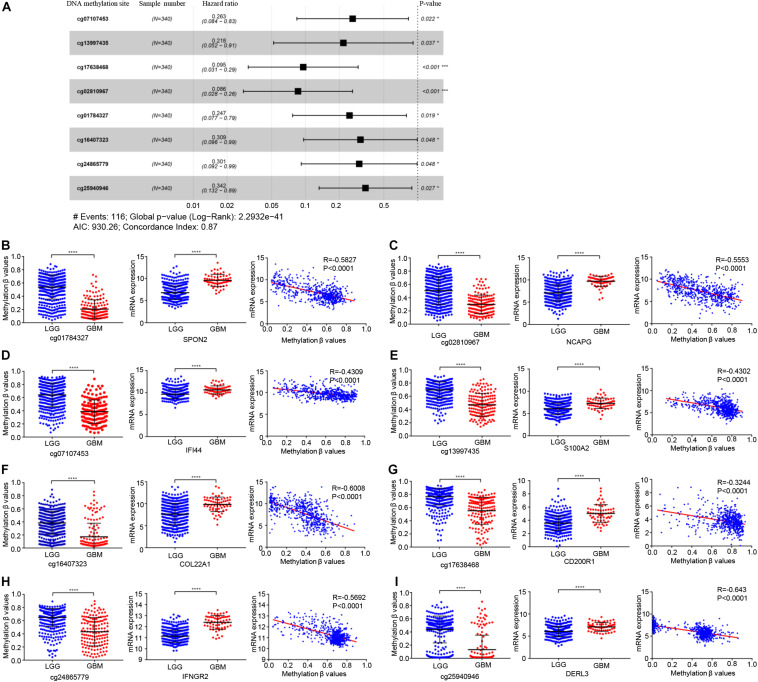
Identification of key prognostic DNA methylation sites. **(A)** The forest plot of the hazard ratio of eight DNA methylation sites. DNA methylation degree of eight methylation sites and corresponding gene expression in low-grade glioma and GBM, and the correlation between DNA methylation degree and corresponding gene expression: **(B)** cg01784327 and SPON2, **(C)** cg02810967 and NCAPG, **(D)** cg07107453 and IFI44 **(E)** cg13997435 and S100A2, **(F)** cg16407323 and COL22A1, **(G)** cg17638468 and CD200R1, **(H)** cg24865779 and IFNGR2, **(I)** cg25940946 and DERL3. Abbreviations: ns, *P* > 0.05, *P < 0.05, ***P < 0.001, ****P < 0.0001.

In total, 679 glioma samples with clinical follow-up information were randomly distributed to a training data set (*n* = 340) and a validation data set (*n* = 339). Clinical information including age, sex, *IDH* and *ATRX* mutation status, 1p/19q status, and treatment conditions are summarized in Supplementary Table 1. Statistical methods were used to build a risk prognosis signature in the training set. DNA methylation sites were identified by univariate Cox analysis, and a total of 629 methylation sites were obtained (FDR <0.01). Next, the selected DNA methylation sites were analyzed by LASSO analysis, and 18 key DNA methylation sites were found (Supplementary Figures 1A,B). Finally, eight DNA methylation sites were screened by multivariate Cox regression, stepwise regression, and screening, which can be used to construct an optimal prognosis signature. Information relative to the eight methylation sites is shown in Table 1 and Figure 3A.

**TABLE 1:** The 8 prognosis-associated DNA methylation sites to construct the epigenetic signature.

**Markers**	**Ref Gene**	**Location**	**UCSC RefGene Group**	**Coefficients**	**HR**	**HR.95L**	**HR.95H**	**P value**
cg01784327	SPON2	Chromosome 4	TSS200	−1.39898	0.24685	0.076772	0.79371	0.019
cg02810967	NCAPG	Chromosome 4	TSS1500	−2.45611	0.085768	0.027991	0.26281	< 0.001
cg07107453	IFI44	Chromosome 1	TSS1500	−1.33461	0.263262	0.083798	0.82707	0.022
cg13997435	S100A2	Chromosome 1	TSS200	−1.52516	0.217585	0.052019	0.91012	0.037
cg16407323	COL22A1	Chromosome 8	TSS1500	−1.1744	0.309003	0.096242	0.99211	0.048
cg17638468	CD200R1	Chromosome 3	TSS200	−2.34894	0.095471	0.030917	0.29481	< 0.001
cg24865779	IFNGR2	Chromosome 21	TSS200	−1.20008	0.30117	0.091688	0.98926	0.048
cg25940946	DERL3	Chromosome 22	TSS200	−1.07163	0.342451	0.132182	0.88721	0.027

**FIGURE 4:**
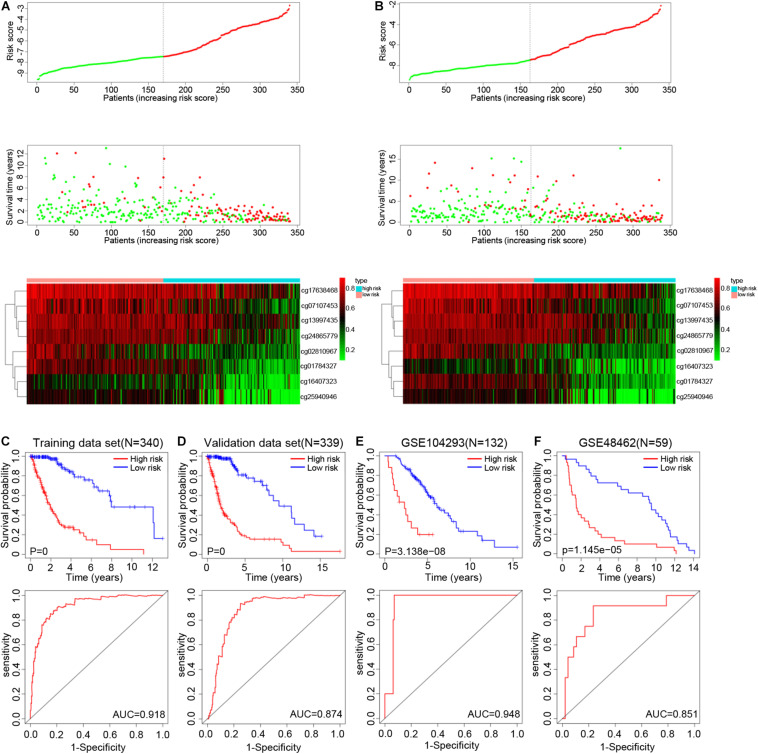
Distribution of the risk score, survival status, and β values of methylation sites in the training set **(A)** and testing set **(B)**. K-M survival curves along with the log-rank test and ROC analysis to evaluate performance of this risk score formula in training set **(C)**, testing set **(D)**, GSE104293 **(E)**, and GSE48462 **(F)**. Abbreviations: K-M, Kaplan-Meier; ROC, receiver operating characteristic; AUC, area under the curve.

The genes corresponding to the eight DNA methylation sites were spondin 2 (*SPON2*), non-SMC condensin I complex subunit G *(NCAPG*), interferon induced protein 44 (*IFI44*), S100 calcium binding protein A2 (*S100A2*), collagen type XXII alpha 1 chain (*COL22A1*), CD200 receptor 1 (*CD200R1*), interferon gamma receptor 2 (*IFNGR2*), and derlin 3 (*DERL3*). The degree of DNA methylation of eight methylation sites and corresponding gene expression in high- and low-risk groups are shown in Figures 3B-I. Risk score = −1.39897577 × β value of cg01784327 – 2.456105464 × β value of cg02810967 – 1.334605549 × β value of cg07107453 – 1.525164679 × β value of cg13997435 −1.174403511 × β value of cg16407323 – 2.348936398 × β value of cg17638468 −1.200080898 × β value of cg24865779 − 1.071625831 × β value of cg25940946. The eight identified CpGs were all positive prognostic factors for gliomas. The distributions of the risk score, survival status, and β values of DNA methylation sites of glioma patients in the training data set and validation data set are shown in Figures 4A,B. K-M analysis showed that there was a significant correlation between the risk score and OS (*p* < 0.001), the AUC was 0.918 (Figure 4C).

In the validation data set, the survival time of high-risk gliomas patient was significantly reduced, with an AUC of 0.874 (Figure 4D). The percentage of high- and low-risk gliomas in different subtypes from TCGA database is shown in Supplementary Table 2 (GBM: Classical, G-CIMP, Mesenchymal, Neural, Proneural; LGG: IDH status, ATRX status, 1p/19q status). In the LGG (GSE104293) and GBM (GSE48462) datasets, the OS of the high-risk group was worse, and the AUC values were 0.948 and 0.851, respectively (Figures 4E,F). In addition, we also analyzed the predictive ability of our epigenetic signature across different statuses of *IDH* mutation, 1p/19q co-deletion, and *ATRX* and *MGMT* methylation states. K-M analysis and ROC curve results showed that the survival rates of high-risk glioma patients were all significantly reduced, which indicated that the model had good independent prognostic ability and reliability (Supplementary Figures 2A-D). The above results indicated that our epigenetic signature has good sensitivity and specificity in predicting the prognosis of glioma patients.

### Association of the Epigenetic Signature With Clinical and Molecular Features in Gliomas

**FIGURE 5:**
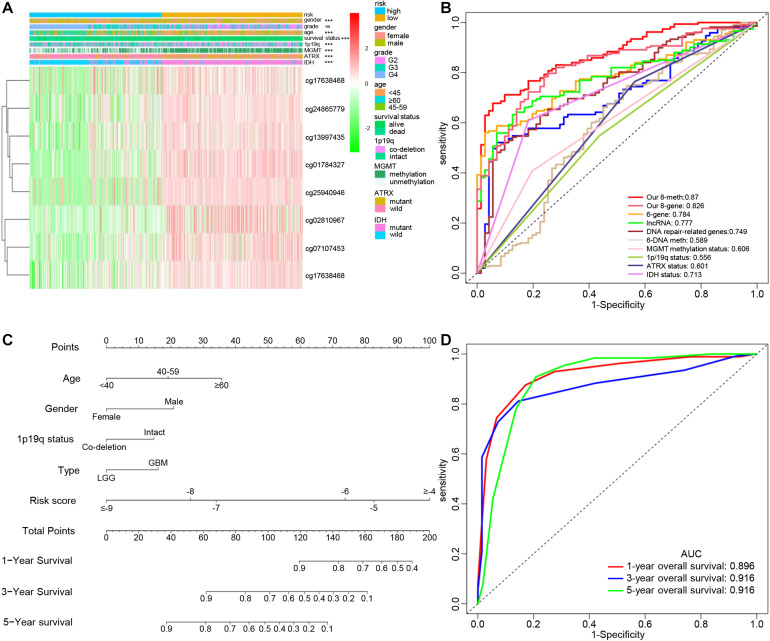
Clinical significance of the epigenetic signature. **(A)** Heatmap view of the methylation degree of eight DNA methylation sites with genomic and clinical characteristics and Chi-square test results. (ns, *P* > 0.05, ***P < 0.001). **(B)** ROC curves showing the sensitivity and specificity of the epigenetic signature and other known biomarkers in predicting the overall survival of glioma patients. **(C)** Development of a nomogram for predicting probabilities of patients with 1-, 3-, and 5-year overall survival. **(D)** ROC curve based on the nomogram for 1-, 3-, and 5-year overall survival probability. Abbreviations: K-M, Kaplan-Meier; ROC, receiver operating characteristic; AUC, area under the curve.

The distribution of clinical and molecular features in high- and low-risk groups and the results of chi-square test are shown in Supplementary Table 3. The heatmap shows the distribution of clinical and molecular features in high- and low-risk groups (Figure 5A). The results showed that except for sex, there were significant differences in age, glioma type, *IDH* mutation status, *ATRX* mutation status, 1p/19q status, MGMT promoter methylation status, and survival status between the high- and low-risk groups. Samples with low risk factors (*MGMT* promoter methylated, mutant-type *IDH*, mutant-type *ATRX*, and 1p/19q co-deletion) had lower risk scores.

### Comparison of the Epigenetic Signature With Other Known Prognostic Biomarkers

With the development of genetics and molecular biology, many molecular biomarkers have been developed for gliomas, such as *IDH* mutation, *ATRX* mutation, *MGMT* methylation status, and 1p/19q status, but they have limitations (Ludwig and Kornblum, 2017). In recent years, numerous prognostic signatures have been developed based on multiple-level molecular data. Zeng et al. (2019) constructed a prognostic model for LGG using DNA damage repair-related genes. Their prognostic model was composed of six genes defined using a weighted gene co-expression network analysis and COX regression analysis (Zhao et al., 2019). In addition to using DNA methylation sites, lncRNAs were also used to construct prognostic models (Yin et al., 2018; Li et al., 2019). Although previous studies have also used DNA methylation sites to build a prognostic risk model, there was no integrated analysis of DNA methylation and gene expression, or use of methylation sites of epigenetic genes to construct a signature. The ROC curve analysis showed that our signature consisting of eight DNA methylation sites had the highest accuracy and was more accurate than the signature composed of the eight corresponding genes alone (Figure 5B).

### Establishment of a Nomogram for the Prognosis of Patients With Glioma

Age, sex, *IDH* mutation status, *ATRX* mutation status, 1p/19q co-deletion status, promoter methylation status of MGMT, radiation therapy history, gliomas type, and risk score were significantly related to OS in the TCGA cohort based on the results from the univariate analysis. Through multivariate analysis of the above factors, age, sex, 1p/19q status, glioma type, and the risk score remained independent and stable prognostic factors (*p* < 0.05) in the cohort (Table 2). A prognostic nomogram based on the independent factors (age, sex, 1p/19q status, gliomas type, and risk score) was constructed (Figure 5C). ROC analysis was performed to evaluate the performance of the nomogram in predicting the prognosis of patients in 1-, 3- and 5-years (Figure 5D).

**TABLE 2:** The univariable and multivariable Cox regression analysis of the 8-DNA methylation signature in glioma patients.

	**Univariate analysis**			**Multivariate analysis**		
**Variables**	**HR**	**95% CI of HR**	**P value**	**HR**	**95% CI of HR**	**P value**
**Age**						
<45	1(Reference)					
45-59	1.9078	1.2691-2.8681	0.001899	1.8819	1.2683-2.7923	0.001687
≥60	3.4931	2.2917-5.3243	6.02e-09	3.5307	2.3423-5.3219	1.69e-09
**Gender**						
Female	1(Reference)					
Male	1.6943	1.2440-2.3076	0.000823	1.7647	1.3119-2.3739	0.000174
**Type**						
LGG	1(Reference)					
GBM	2.4083	1.6645-3.4847	3.12e-06	2.6290	1.8475-3.7411	7.85e-08
**Radiation therapy**						
NO	1(Reference)					
YES	1.1441	0.7716-1.6964	0.502964			
**IDH status**						
Wild	1(Reference)					
Mutant	0.7883	0.4617-1.3459	0.383487			
**ATRX status**						
Wild	1(Reference)					
Mutant	1.1234	0.7305-1.7274	0.596238			
**the methylation status of MGMT promoter**						
unmethylated	1(Reference)					
methylated	0.7807	0.5703-1.0687	0.122225			
**1p/19q status**						
intact	1(Reference)			1(Reference)		
Co-deletion	0.5915	0.4339-0.8062	0.000891	0.5362	0.4023-0.7148	2.14e-05
**Risk Score**						
Low	1(Reference)			1(Reference)		
High	3.2527	1.9617-5.3932	4.84e-06	4.0223	2.7383-5.9084	1.30e-12

### Comparison of the Results of the GSEA Analysis Between High- and Low-Risk Groups

**FIGURE 6:**
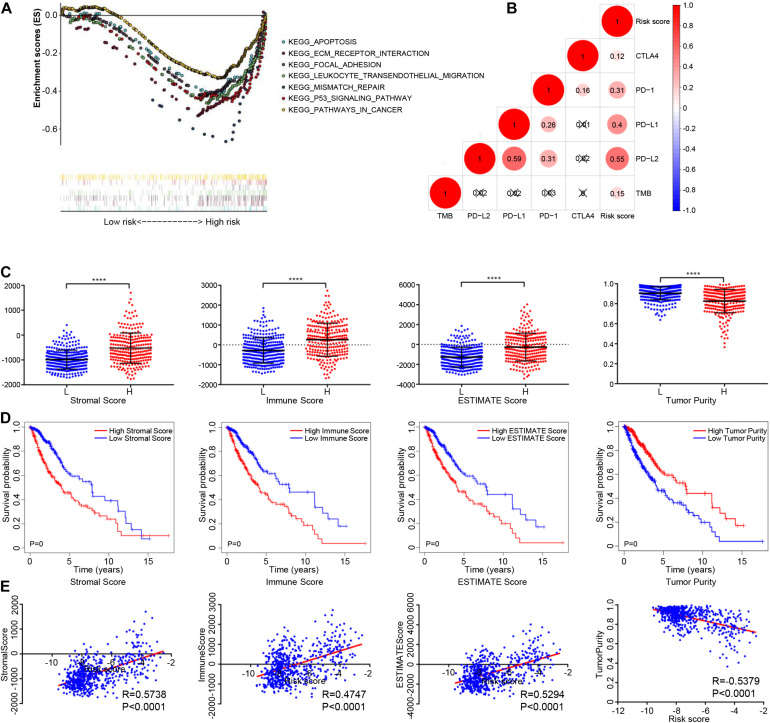
The relationship between the epigenetic signature and immune cell infiltration. **(A)** GSEA showing the differences between high- and low-risk groups. **(B)** Correlation analysis between the risk score and immune checkpoint expression. **(C)** Distribution of stromal scores, immune score, ESTIMATE score, and tumor purity among high- and low-risk glioma patients. **(D)** Glioma patients were divided into two groups based on Stromal scores, immune score, ESTIMATE score, and tumor purity. K-M survival curve show overall survival of the low score group is longer than high score group, as indicated by the log-rank test. **(E)** Correlation analysis between the risk score and stromal scores, immune score, ESTIMATE score, and tumor purity. Abbreviations: K-M, Kaplan-Meier; ns, *P* > 0.05, ****P < 0.0001.

We used GSEA analysis to compare the KEGG pathways involved in high- and low-risk groups, the results showed that mismatch repair, focal adhesion, leukocyte transendothelial migration, apoptosis, pathways in cancer, and the p53 signaling pathway were significantly enriched in the high-risk group (Figure 6A).

### Associations Between the Epigenetic Signature and Immune-Checkpoint Blockade Immunotherapy-Related Signature

To study the potential role of the epigenetic signature in immune-checkpoint blockade (ICB) immunotherapy, correlation analysis was carried out with these known immunotherapy-related signatures. The results showed that the epigenetic signature was positively correlated with expression of PD-1, PD-L1, PD-L2, CTLA-4, and TMB (Figure 6B). In addition, we also analyzed the correlation between the eight methylation sites and immunotherapy-related signatures, as well as the correlation between the eight genes and immunotherapy-related signatures, as shown in Supplementary Figures 3A,B. In summary, although our eight-DNA methylation prognostic signature was constructed to accurately predict the prognosis of gliomas patients, it may also play a potential role in defining the application of ICB immunotherapy for glioma patients.

### Epigenetic Signature Was Associated With the Tumor Microenvironment

The immune score, stromal score, and ESTIMATE score in the high-risk group were higher than those in the low-risk group, while the tumor purity in the high-risk group was lower than that in the low-risk group (Figure 6C). K-M analysis showed that a poorer prognosis was associated with higher immune scores, stromal scores, ESTIMATE scores, and lower tumor purity (Figure 6D). The Immune, stromal, and ESTIMATE scores were positively correlated with the risk score, while tumor purity was negatively correlated with risk score (| correlation| > 0.20, *P* < 0.05), as shown in Figure 6E. In addition, we analyzed the association between the eight methylation sites, their corresponding genes, and the immune, stromal, and ESTIMATE scores, and tumor purity (Supplementary Figures 4A,B).

### Relationship Between the Epigenetic Signature and the Proportion of Immune Cell Infiltration

**FIGURE 7:**
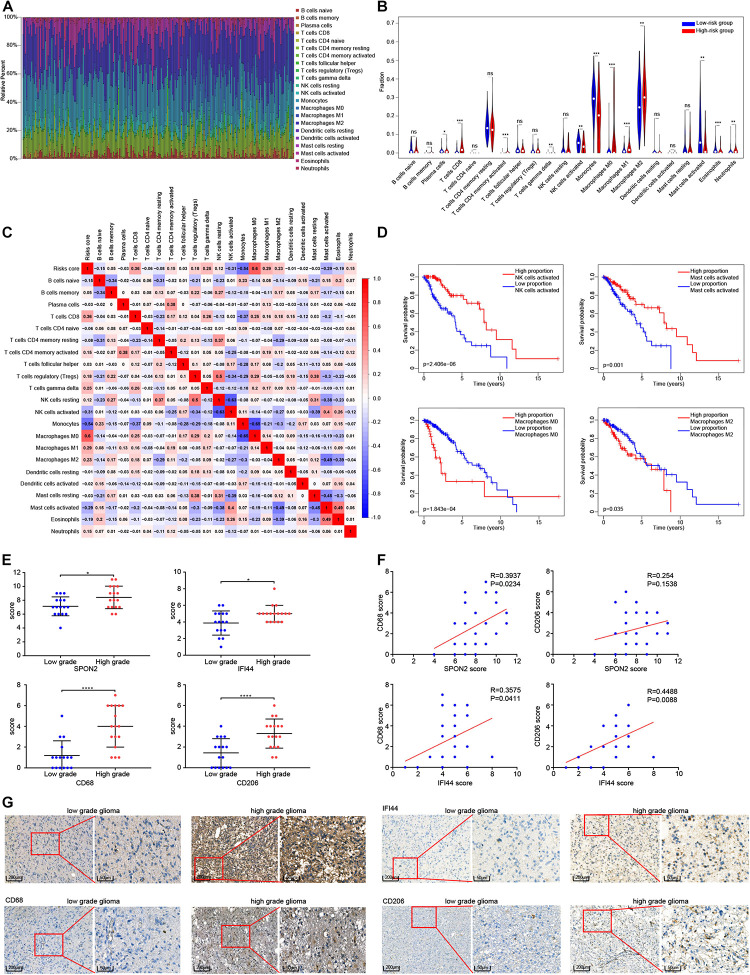
Analysis of the relationship between risk score and immune cell subtypes. **(A)** Proportion of immune cells in each glioma sample are indicated with different colors, and lengths of the bars in the bar chart indicate the levels of the immune cell proportions. **(B)** Comparison of the proportion of 22 immune cell types in high and low risk groups. **(C)** Correlation risk score and the proportion of 22 immune cell types. **(D)** Survival curves of four immune cell types significantly related to the prognosis of patients with glioma. **(E)** Comparison of the scoring of SPON2, IFI44, CD68 and CD206 expression in low-and high-grade gliomas tissues. **(F)** Correlation analysis between the scoring of SPON2, IFI44, CD68 and CD206 expression. **(G)** Representative immunohistochemistry of low- and high-grade gliomas tissues with the scoring of SPON2, IFI44, CD68 and CD206 expression. Abbreviations: ns, *P* > 0.05, *P < 0.05, **P < 0.01, ***P < 0.001, ****P < 0.0001.

After samples with *P* < 0.05 were excluded, the remaining 216 samples were subjected to CIBERSORT analysis, including 192 LGG samples and 24 GBM samples. Twenty-two subtypes of immune cells were obtained (Figure 7A). A *t*-test was used to compare differences between the 22 kinds between the immune cell types in the high-risk and low-risk groups, as shown in Figure 7B. The proportion of CD8+ T cells, CD4+ memory activated T cells, gamma delta T cells, M0 macrophages, M1 macrophages, M2 macrophages, and neutrophils were higher in high-risk group, while plasma cells, activated NK cells, monocytes, activated mast cells, and eosinophils were higher in the low-risk group (*P* < 0.05). Pearson’s correlation analysis was used to analyze the correlation between the risk score and the different immune cell proportions. CD8+ T cells, CD4+ memory activated T cells, gamma delta T cells, M0 macrophages, M1 macrophages, and M2 macrophages were positively correlated with risk score, while activated NK cells, monocytes, activated mast cells were negatively correlated with risk score (| correlation| > 0.20, *P* < 0.05), as shown in Figure 7C. The results of the K-M analysis showed that glioma patients with a low proportion of activated NK cells, activated Mast cells and high percentage of M0 macrophages and M1 macrophages had a shorter OS (Figure 7D). Finally, we analyzed the correlation between the methylation degree of eight DNA methylation sites and the proportion 22 immune cell subtypes, and the correlation between the expression of the eight corresponding genes and the proportion of the immune cell subtypes (Supplementary Figures 5A,B).

### Immunostaining of SPON2, IFI44, CD68, and CD206 Expression in Glioma Tissue Samples

We performed immunohistochemical staining of SPON2, IFI44, CD68, and CD206 in 33 cases of gliomas. The immunostaining score evaluated by two professional pathologists showed that SPON2, IFI44, CD68, and CD206 were significantly overexpressed in high-grade gliomas (Figure 7E,G). Notably, the immunostaining score of SPON2 was positively correlated with the macrophage marker CD68, but not with the M2 macrophage marker CD206. The immunostaining score of IFI44 was positively correlated with CD68 and CD206 expression (Figure 7F). These results suggested that SPON2 may be involved in the recruitment of non-M2 macrophages, while IFI44 may participate in the recruitment of M2 macrophages.

## Discussion

Glioma is the most common malignant tumor of the central nervous system, accounting for about 80% of diagnosed cases. Despite the implementation of surgical treatment and adjuvant therapy, the prognosis of patients with glioma is still poor, resulting difficulty to cure and death (Hemmati et al., 2003). Investigating the differences between GBM and LGG can improve the understanding of the glioma development and the development of treatment strategies of brain tumors (Ceccarelli et al., 2016; Binder et al., 2019). Risk classification of gliomas patients is helpful to guide the choice of treatment, while molecular markers have the advantages of providing prognostic information and exploring the mechanism of tumor progression. We integrated multiple DNA methylation sites to construct a more sensitive and specific signature. Using the methylation sites of the screened epigenetic difference genes, a prognostic signature based on eight methylation sites was developed by applying a series of statistical approaches. K-M analysis showed that the OS of high- and low-risk groups could be easily stratified, and ROC analysis verified that the signature had accurate predictive ability. The clinical molecular characteristics and risk scores were analyzed using Cox regression analysis. The prognosis of GBM, older age, male sex, absence of 1p/19q co-deletion, and a high-risk score for gliomas showed worse prognosis, and all these features were independent prognostic factors (HR > 1 and *P* < 0.05). We used the above factors to construct a nomogram able to predict the 1-, 3-, and 5-year prognosis of patients with glioma.

There are more immunosuppressive factors than pro-inflammatory factors expressed in gliomas, suggesting that the microenvironment of gliomas is mainly immunosuppressive (Sokratous et al., 2017). Monocytes from the peripheral blood infiltrate to form microglia-like cells, which participate in the renewal of microglia. Under different signal stimulation conditions, microglia/macrophages M0 in gliomas can develop into M1 microglia/macrophages that promote inflammation and inhibit tumor growth, or M2 microglia/macrophages that inhibit inflammatory response and promote tumor growth, with mainly the infiltration of the latter and namely tumor associated macrophages (TAM) (Durafourt et al., 2012; Shapouri-Moghaddam et al., 2018). Although the tumor immune microenvironment plays an important role in tumor initiation and development, there is no effective signature available that evaluates the immune infiltration of gliomas. In addition, the epigenetic signature has shown limited value in the exploration or evaluation of tumor immune subtypes, especially in the immune infiltration (Aichmüller et al., 2020; Wu et al., 2020).

We integrated publicly available gene expression and DNA methylation data to define epigenetic regulatory mechanisms of some genes essential for immune infiltration. Further, this study has emphasized the potential relationship between CpG methylation markers and indicators of immune infiltration in gliomas. The Infinium Human Methylation450 BeadChip can detected more than 450,000 CpG sites, which may serve as a more suitable alternative marker of immune infiltration. To investigate the correlation between our signature and immune infiltration, we used the following approach. First, the GSEA findings revealed that leukocyte transendothelial migration was significantly enriched in the high-risk group. Second, the ESTIMATE algorithm was used to evaluate the degree of immune and stromal cell infiltration and tumor purity of each glioma patient. Pearson’s correlation analysis showed that the risk score was positively correlated with the degree of immune cell and stromal cell infiltration, and negatively correlated with tumor purity. Third, the proportion of 22 immune cells subtypes in each glioma patient was evaluated using the CIBERSORT algorithm. Pearson correlation analysis revealed that the risk score was positively correlated with the infiltration of CD8+ T cells, gamma delta T cells, M0 macrophages, M1 macrophages, and M2 macrophages and was negatively correlated with the proportion of activated NK cells, monocytes, and activated mast cells. The high proportion of M0 macrophages and M2 macrophages was negatively correlated with patient prognosis, whereas a high proportion of NK cells and activated mast cells was positively correlated with prognosis.

NK cells have been reported to exert their cytotoxicity by secreting tumor necrosis factor (TNF) and interferon (IFN) to kill susceptible target cells (Moretta and Moretta, 2004; Raulet, 2004). The possible mechanism of mast cells inhibiting tumor are related to the immune response, but the specific roles of their intracellular bioactive cytokines in tumorigenesis and progression is still difficult to determine. Many experimental results have suggested that mast cells can secrete a variety of cytokines, such as TNF-α, interleukin (IL)-3, IL-4, IL-6, and IL-8 (Gordon et al., 1990; Burd et al., 1995). According to our analysis, it can be inferred that NK cells and mast cells can clear tumor tissue through immune activity. In brief, the epigenetic signature can stratify glioma patients into different immune infiltration subgroups (“low immune infiltration type” and “high immune infiltration type”). Therefore, this novel signature may reflect the immune microenvironment and serves as a prognostic marker in gliomas. We also found that the type of immune infiltration of gliomas was related to the status of *IDH* mutation or 1p/19q co-deletion. Gliomas with mutated *IDH* or 1p/19q co-deletion presented a lower degree of immune infiltration and better prognosis.

Amankulor et al. (2017) found that *IDH* mutation significantly reduced the infiltration of immune cells such as macrophages, microglia, monocytes, and neutrophils, resulting in the suppression of the tumor-associated immune system. However, it is not clear whether the effect of 1p/19q co-deletion status on prognosis is mediated by immune regulation, which is worthy of further study. In addition, we verified the association of SPON2 and IFI44 genes with macrophage recruitment and performed immunohistochemical staining on glioma samples for SPON2, IFI44, the macrophage marker CD68, and the M2 macrophage marker CD206. SPON2, IFI44, CD68, and CD206 were highly expressed in high-grade gliomas, and SPON2 was positively correlated with CD68 rather than CD206, whereas IFI44 was positively correlated with both CD68 and CD206 expression. Recent studies have found that SPON2, a member of the Mindin F-pondin family of conserved secretory ECM proteins, plays an important role in immunity by participating in the initiation of the immune response, and acts as an integrin ligand for inflammatory cell recruitment and T cell priming (Jia et al., 2005; Li et al., 2006). Combined with our experimental results, SPON2 may be involved in the recruitment of non-M2 macrophages in glioma. IFI44 gene, whose expression is induced by alpha/beta-IFN, can block extracellular signal-regulated kinase signals, and causes cell cycle arrest by binding to intracellular GTP (Kitamura et al., 1994; Hallen et al., 2007). Further, IFI44, as an immune-related gene, is involved in immune diseases, but the studies in tumors is insufficient (DeDiego et al., 2019). Our study showed that IFI44 was positively correlated with macrophage infiltration in gliomas. The potential role of IFI44 in tumor formation and development needs further experimental verification.

Previous studies have shown that CD200R1 is also an immune-related gene that encodes the OX-2 membrane glycoprotein receptor, which is mainly distributed on the surface of myeloid cells and T lymphocytes (Gorczynski, 2005). CD200 interacts with its receptor CD200R1 to trigger immunosuppressive signals, resulting in macrophage suppression, regulatory T cell induction, cytokine profile switching from Th1 to Th2, and finally the inhibition tumor-specific T-cell immunity (Gorczynski et al., 1999; Vaine and Soberman, 2014). IFN-γ binds to the IFN-γ receptor (heterodimer composed of IFNGR1 and IFNGR2) and activates a downstream signal pathway that can inhibit tumorigenesis and immunomodulatory effects (Parker et al., 2016; Lin et al., 2017). IFN-γ induces the polarization of M1 macrophages, but not M2 macrophages (Duluc et al., 2009). However, it has not been reported whether the other four genes, NCAPG, S100A2, DERL3, and COL22A1, exert any biological role in the interaction between tumor and immune cells. In the future, we will perform additional experiments to verify the biological function in gliomas of the eight genes identified in this study, which may provide additional treatment targets and improve patient management.

Finally, we studied the monitoring role of DNA methylation biomarkers in ICB immunotherapy. The epigenetic signature was also highly correlated with the expression of CTLA4, PD-L1, and PD-1, suggesting that it may be a potential indicator of cancer immune infiltration and may predict the patients’ response to immunotherapy drugs. An individualized treatment regimen can be realized through the stratification of patients, which will significantly improve the effects of immunotherapy and prolong the survival of patients.

In conclusion, this study highlighted the potential role of DNA methylation in assessing risk prognosis and monitoring immune infiltration. Our research innovatively used an approach based on an epigenetic signature to classify gliomas into two categories: the “low immune infiltration type” and “high immune infiltration type”. In addition, the eight genes in the signature may be involved in glioma progression and may have a significant potential to improve risk stratification, and regulate immune cell infiltration, and ICB immunotherapy response. If further verified, the epigenetic signature may improve the stratification and immunotherapy options of cancer patients in clinical trials.

## Data Availability Statement

’The datasets presented in this study can be found in online repositories. The names of the repository/repositories and accession number(s) can be found in the article/Supplementary Material.

## Ethics Statement

The studies involving human participants were reviewed and approved by the Ethics Committee of the Affiliated Cancer Hospital of Guangzhou Medical University. Written informed consent to participate in this study was provided by the participants’ legal guardian/next of kin.

## Author Contributions

JZ and GZ: conception and design. GZ and QZ: administrative support. JY, LL, YS, and DZ: provision of study materials or patients. JY, DH, and GW: collection and assembly of data. JZ, NX, and MY: data analysis and interpretation. All authors are final approval of manuscript.

## Conflict of Interest

The authors declare that the research was conducted in the absence of any commercial or financial relationships that could be construed as a potential conflict of interest.

## Footnotes

1 https://portal.gdc.cancer.gov/

2 https://www.ncbi.nlm.nih.gov/geo/

3 http://www.bioconductor.org/packages/release/bioc/html/edgeR.html

4 http://www.bioconductor.org/packages/release/bioc/html/limma.html

5 https://david.ncifcrf.gov/

6 https://CRAN.R-project.org/package=caret

7 https://CRAN.R-project.org/package=glmnet

8 https://CRAN.R-project.org/package=rms

## References

[B1] Aichmüller C. Iskar M. Jones D. Korshunov A. Radlwimmer B. Kool M. (2020). Pilocytic astrocytoma demethylation and transcriptional landscapes link bZIP transcription factors to immune response. *Neuro Oncol.* 22 1327–1338. 10.1093/neuonc/noaa035 32052037 PMC7523467

[B2] Amankulor N. Kim Y. Arora S. Kargl J. Szulzewsky F. Hanke M. (2017). Mutant IDH1 regulates the tumor-associated immune system in gliomas. *Genes Dev.* 31 774–786. 10.1101/gad.294991.116 28465358 PMC5435890

[B3] Bindea G. Mlecnik B. Tosolini M. Kirilovsky A. Waldner M. Obenauf A. (2013). Spatiotemporal dynamics of intratumoral immune cells reveal the immune landscape in human cancer. *Immunity* 39 782–795. 10.1016/j.immuni.2013.10.003 24138885

[B4] Binder H. Willscher E. Loeffler-Wirth H. Hopp L. Jones D. Pfister S. (2019). DNA methylation, transcriptome and genetic copy number signatures of diffuse cerebral WHO grade II/III gliomas resolve cancer heterogeneity and development. *Acta Neuropathol. Commun.* 7:59. 10.1186/s40478-019-0704-8 31023364 PMC6482573

[B5] Buckner J. Shaw E. Pugh S. Chakravarti A. Gilbert M. Barger G. (2016). Radiation plus procarbazine, CCNU, and vincristine in low-grade glioma. *N. Engl. J. Med.* 374 1344–1355. 10.1056/NEJMoa1500925 27050206 PMC5170873

[B6] Burd P. Thompson W. Max E. Mills F. (1995). Activated mast cells produce interleukin 13. *J. Exp. Med.* 181 1373–1380. 10.1084/jem.181.4.1373 7535336 PMC2191950

[B7] Ceccarelli M. Barthel F. Malta T. Sabedot T. Salama S. Murray B. (2016). Molecular profiling reveals biologically discrete subsets and pathways of progression in diffuse Glioma. *Cell* 164 550–563. 10.1016/j.cell.2015.12.028 26824661 PMC4754110

[B8] Cooper L. Gutman D. Chisolm C. Appin C. Kong J. Rong Y. (2012). The tumor microenvironment strongly impacts master transcriptional regulators and gene expression class of glioblastoma. *Am. J. Pathol.* 180 2108–2119. 10.1016/j.ajpath.2012.01.040 22440258 PMC3354586

[B9] Crommentuijn M. Schetters S. Dusoswa S. Kruijssen L. Garcia-Vallejo J. van K. (2020). Immune involvement of the contralateral hemisphere in a glioblastoma mouse model. *J. Immunother. Cancer* 8:e000323. 10.1136/jitc-2019-000323 32303613 PMC7204813

[B10] DeDiego M. Nogales A. Martinez-Sobrido L. Topham D. (2019). Interferon-induced protein 44 interacts with cellular FK506-binding protein 5, negatively regulates host antiviral responses, and supports virus replication. *mBio* 10:e01839-19. 10.1128/mBio.01839-19 31455651 PMC6712396

[B11] Duluc D. Corvaisier M. Blanchard S. Catala L. Descamps P. Gamelin E. (2009). Interferon-gamma reverses the immunosuppressive and protumoral properties and prevents the generation of human tumor-associated macrophages. *Int. J. Cancer* 125 367–373. 10.1002/ijc.24401 19378341

[B12] Durafourt B. Moore C. Zammit D. Johnson T. Zaguia F. Guiot M. (2012). Comparison of polarization properties of human adult microglia and blood-derived macrophages. *Glia* 60 717–727. 10.1002/glia.22298 22290798

[B13] Fleischer T. Frigessi A. Johnson K. Edvardsen H. Touleimat N. Klajic J. (2014). Genome-wide DNA methylation profiles in progression to in situ and invasive carcinoma of the breast with impact on gene transcription and prognosis. *Genome Biol.* 15:435. 10.1186/preaccept-2333349012841587 25146004 PMC4165906

[B14] Gorczynski L. Chen Z. Hu J. Kai Y. Lei J. Ramakrishna V. (1999). Evidence that an OX-2-positive cell can inhibit the stimulation of type 1 cytokine production by bone marrow-derived B7-1 (and B7-2)-positive dendritic cells. *J. Immunol.* 162 774–781. 9916698

[B15] Gorczynski R. M. (2005). CD200 and its receptors as targets for immunoregulation. *Curr. Opin. Investig. Drugs* 6 483–488. 15912961

[B16] Gordon J. Burd P. Galli S. (1990). Mast cells as a source of multifunctional cytokines. *Immunol. Today* 11 458–464. 10.1016/0167-5699(90)90176-a 2073318

[B17] Hallen L. Burki Y. Ebeling M. Broger C. Siegrist F. Oroszlan-Szovik K. (2007). Antiproliferative activity of the human IFN-alpha-inducible protein IFI44. *J. Interferon Cytokine Res.* 27 675–680. 10.1089/jir.2007.0021 17784819

[B18] Hanif F. Muzaffar K. Perveen K. Malhi S. Simjee S. (2017). Glioblastoma multiforme: a review of its epidemiology and pathogenesis through clinical presentation and treatment. *Asian Pac. J. Cancer Prev.* 18 3–9. 10.22034/apjcp.2017.18.1.3 28239999 PMC5563115

[B19] Hemmati H. Nakano I. Lazareff J. Masterman-Smith M. Geschwind D. Bronner-Fraser M. (2003). Cancerous stem cells can arise from pediatric brain tumors. *Proc. Natl. Acad. Sci. U.S.A.* 100 15178–15183. 10.1073/pnas.2036535100 14645703 PMC299944

[B20] Issa J. (2012). DNA methylation as a clinical marker in oncology. *J. Clin. Oncol.* 30 2566–2568. 10.1200/jco.2012.42.1016 22564986

[B21] Jia W. Li H. He Y. W. (2005). The extracellular matrix protein mindin serves as an integrin ligand and is critical for inflammatory cell recruitment. *Blood* 106 3854–3859. 10.1182/blood-2005-04-1658 16105980 PMC1895097

[B22] Kitamura A. Takahashi K. Okajima A. Kitamura N. (1994). Induction of the human gene for p44, a hepatitis-C-associated microtubular aggregate protein, by interferon-alpha/beta. *Eur. J. Biochem.* 224 877–883. 10.1111/j.1432-1033.1994.00877.x 7925411

[B23] Klutstein M. Nejman D. Greenfield R. Cedar H. (2016). DNA methylation in cancer and aging. *Cancer Res.* 76 3446–3450. 10.1158/0008-5472.CAN-15-3278 27256564

[B24] Ladomersky E. Zhai L. Lauing K. Bell A. Xu J. Kocherginsky M. (2020). Advanced age increases immunosuppression in the brain and decreases immunotherapeutic efficacy in subjects with glioblastoma. *Clin. Cancer Res.* 26 5232–5245. 10.1158/1078-0432.Ccr-19-3874 32546647 PMC7541490

[B25] Li D. Lu J. Li H. Qi S. Yu L. (2019). Identification of a long noncoding RNA signature to predict outcomes of glioblastoma. *Mol. Med. Rep.* 19 5406–5416. 10.3892/mmr.2019.10184 31059035 PMC6522932

[B26] Li H. Oliver T. Jia W. He Y. W. (2006). Efficient dendritic cell priming of T lymphocytes depends on the extracellular matrix protein mindin. *EMBO J.* 25 4097–4107. 10.1038/sj.emboj.7601289 16917498 PMC1560362

[B27] Lin C. F. Lin C. M. Lee K. Y. Wu S. Y. Feng P. H. Chen K. Y. (2017). Escape from IFN-gamma-dependent immunosurveillance in tumorigenesis. *J. Biomed. Sci.* 24:10. 10.1186/s12929-017-0317-0 28143527 PMC5286687

[B28] Louis D. Perry A. Reifenberger G. von Deimling A. Figarella-Branger D. Cavenee W. (2016). The 2016 World Health Organization classification of tumors of the central nervous system: a summary. *Acta Neuropathol. Commun.* 131 803–820. 10.1007/s00401-016-1545-1 27157931

[B29] Ludwig K. Kornblum H. (2017). Molecular markers in glioma. *J. Neurooncol.* 134 505–512. 10.1007/s11060-017-2379-y 28233083 PMC5568999

[B30] Malzkorn B. Wolter M. Riemenschneider M. Reifenberger G. (2011). Unraveling the glioma epigenome: from molecular mechanisms to novel biomarkers and therapeutic targets. *Brain Pathol.* 21 619–632. 10.1111/j.1750-3639.2011.00536.x 21939466 PMC8094062

[B31] Moretta L. Moretta A. (2004). Unravelling natural killer cell function: triggering and inhibitory human NK receptors. *EMBO J.* 23 255–259. 10.1038/sj.emboj.7600019 14685277 PMC1271745

[B32] Newman A. Liu C. Green M. Gentles A. Feng W. Xu Y. (2015). Robust enumeration of cell subsets from tissue expression profiles. *Nat. Med.* 12 453–457. 10.1038/nmeth.3337 25822800 PMC4739640

[B33] O’Day S. Maio M. Chiarion-Sileni V. Gajewski T. Pehamberger H. Bondarenko I. (2010). Efficacy and safety of ipilimumab monotherapy in patients with pretreated advanced melanoma: a multicenter single-arm phase II study. *Ann. Oncol.* 21 1712–1717. 10.1093/annonc/mdq013 20147741

[B34] Omuro A. Vlahovic G. Lim M. Sahebjam S. Baehring J. Cloughesy T. (2018). Nivolumab with or without ipilimumab in patients with recurrent glioblastoma: results from exploratory phase I cohorts of CheckMate 143. *Neuro Oncol.* 20 674–686. 10.1093/neuonc/nox208 29106665 PMC5892140

[B35] Ostrom Q. Cioffi G. Gittleman H. Patil N. Waite K. Kruchko C. (2019). CBTRUS statistical report: primary brain and other central nervous system tumors diagnosed in the United States in 2012-2016. *Neuro Oncol.* 21 v1–v100. 10.1093/neuonc/noz150 31675094 PMC6823730

[B36] Pan Y. Liu G. Zhou F. Su B. Li Y. (2018). DNA methylation profiles in cancer diagnosis and therapeutics. *Clin. Exp. Med.* 18 1–14. 10.1007/s10238-017-0467-0 28752221

[B37] Parker B. S. Rautela J. Hertzog P. J. (2016). Antitumour actions of interferons: implications for cancer therapy. *Nat. Rev. Cancer* 16 131–144. 10.1038/nrc.2016.14 26911188

[B38] Raulet D. (2004). Interplay of natural killer cells and their receptors with the adaptive immune response. *Nat. Immunol.* 5 996–1002. 10.1038/ni1114 15454923

[B39] Ritchie M. Phipson B. Wu D. Hu Y. Law C. Shi W. (2015). limma powers differential expression analyses for RNA-sequencing and microarray studies. *Nucleic Acids Res.* 43:e47. 10.1093/nar/gkv007 25605792 PMC4402510

[B40] Rizvi N. Mazières J. Planchard D. Stinchcombe T. Dy G. Antonia S. (2015). Activity and safety of nivolumab, an anti-PD-1 immune checkpoint inhibitor, for patients with advanced, refractory squamous non-small-cell lung cancer (CheckMate 063): a phase 2, single-arm trial. *Lancet Oncol.* 16 257–265. 10.1016/s1470-2045(15)70054-9 25704439 PMC5726228

[B41] Shapouri-Moghaddam A. Mohammadian S. Vazini H. Taghadosi M. Esmaeili S. Mardani F. (2018). Macrophage plasticity, polarization, and function in health and disease. *J. Cell Physiol.* 233 6425–6440. 10.1002/jcp.26429 29319160 PMC13482560

[B42] Simon N. Friedman J. Hastie T. Tibshirani R. (2011). Regularization Paths for Cox’s proportional Hazards model via coordinate descent. *J. Stat. Softw.* 39 1–13. 10.18637/jss.v039.i05 27065756 PMC4824408

[B43] Sokratous G. Polyzoidis S. Ashkan K. (2017). Immune infiltration of tumor microenvironment following immunotherapy for glioblastoma multiforme. *Hum. Vaccin. Immunother.* 13, 2575–2582. 10.1080/21645515.2017.1303582 28362548 PMC5703406

[B44] Subramanian A. Tamayo P. Mootha V. Mukherjee S. Ebert B. Gillette M. (2005). Gene set enrichment analysis: a knowledge-based approach for interpreting genome-wide expression profiles. *Proc. Natl. Acad. Sci. U.S.A.* 102 15545–15550. 10.1073/pnas.0506580102 16199517 PMC1239896

[B45] Timp W. Bravo H. McDonald O. Goggins M. Umbricht C. Zeiger M. (2014). Large hypomethylated blocks as a universal defining epigenetic alteration in human solid tumors. *Genome Med.* 6:61. 10.1186/s13073-014-0061-y 25191524 PMC4154522

[B46] Vaine C. A. Soberman R. J. (2014). The CD200-CD200R1 inhibitory signaling pathway: immune regulation and host-pathogen interactions. *Adv. Immunol.* 121 191–211. 10.1016/B978-0-12-800100-4.00005-2 24388216 PMC4617684

[B47] van den Bent M. (2014). Practice changing mature results of RTOG study 9802: another positive PCV trial makes adjuvant chemotherapy part of standard of care in low-grade glioma. *Neuro Oncol.* 16 1570–1574. 10.1093/neuonc/nou297 25355680 PMC4232091

[B48] van den Bent M. Brandes A. Taphoorn M. Kros J. Kouwenhoven M. Delattre J. (2013). Adjuvant procarbazine, lomustine, and vincristine chemotherapy in newly diagnosed anaplastic oligodendroglioma: long-term follow-up of EORTC brain tumor group study 26951. *J. Clin. Oncol.* 31 344–350. 10.1200/jco.2012.43.2229 23071237

[B49] Wu Y. Fletcher M. Gu Z. Wang Q. Costa B. Bertoni A. (2020). Glioblastoma epigenome profiling identifies SOX10 as a master regulator of molecular tumour subtype. *Nat. Commun.* 11:6434. 10.1038/s41467-020-20225-w 33339831 PMC7749178

[B50] Xiong Y. Wang K. Zhou H. Peng L. You W. Fu Z. (2018). Profiles of immune infiltration in colorectal cancer and their clinical significant: a gene expression-based study. *Cancer Med.* 7 4496–4508. 10.1002/cam4.1745 30117315 PMC6144159

[B51] Yi L. Xiao H. Xu M. Ye X. Hu J. Li F. (2011). Glioma-initiating cells: a predominant role in microglia/macrophages tropism to glioma. *J. Neuroimmunol.* 232 75–82. 10.1016/j.jneuroim.2010.10.011 21056915

[B52] Yin A. A. Lu N. Etcheverry A. Aubry M. Barnholtz-Sloan J. Zhang L. H. (2018). A novel prognostic six-CpG signature in glioblastomas. *CNS Neurosci. Ther.* 24 167–177. 10.1111/cns.12786 29350455 PMC6489960

[B53] Yoshihara K. Shahmoradgoli M. Martínez E. Vegesna R. Kim H. Torres-Garcia W. (2013). Inferring tumour purity and stromal and immune cell admixture from expression data. *Nat. Commun.* 4:2612. 10.1038/ncomms3612 24113773 PMC3826632

[B54] Zeng F. Liu X. Wang K. Zhao Z. Li G. (2019). Transcriptomic profiling identifies a DNA repair-related signature as a novel prognostic marker in lower grade Gliomas. *Cancer Epidemiol. Biomarkers Prev.* 28 2079–2086. 10.1158/1055-9965.EPI-19-0740 31533943

[B55] Zhao J. Wang L. Hu G. Wei B. (2019). A 6-gene risk signature predicts survival of Glioblastoma multiforme. *Biomed. Res. Int.* 2019:1649423. 10.1155/2019/1649423 31531345 PMC6720050

[B56] Zhu J. Yao X. (2009). Use of DNA methylation for cancer detection: promises and challenges. *Int. J. Biochem. Cell Biol.* 41 147–154. 10.1016/j.biocel.2008.09.003 18834953

