# Supplementary figures and images for: Integrative Analysis of DNA Methylation and Transcriptome Identifies a Predictive Epigenetic Signature Associated With Immune Infiltration in Gliomas
Source: Front Cell Dev Biol. 2021 May 31;9:670854. doi: 10.3389/fcell.2021.670854 (PMC8203203; doi:10.3389/fcell.2021.670854)

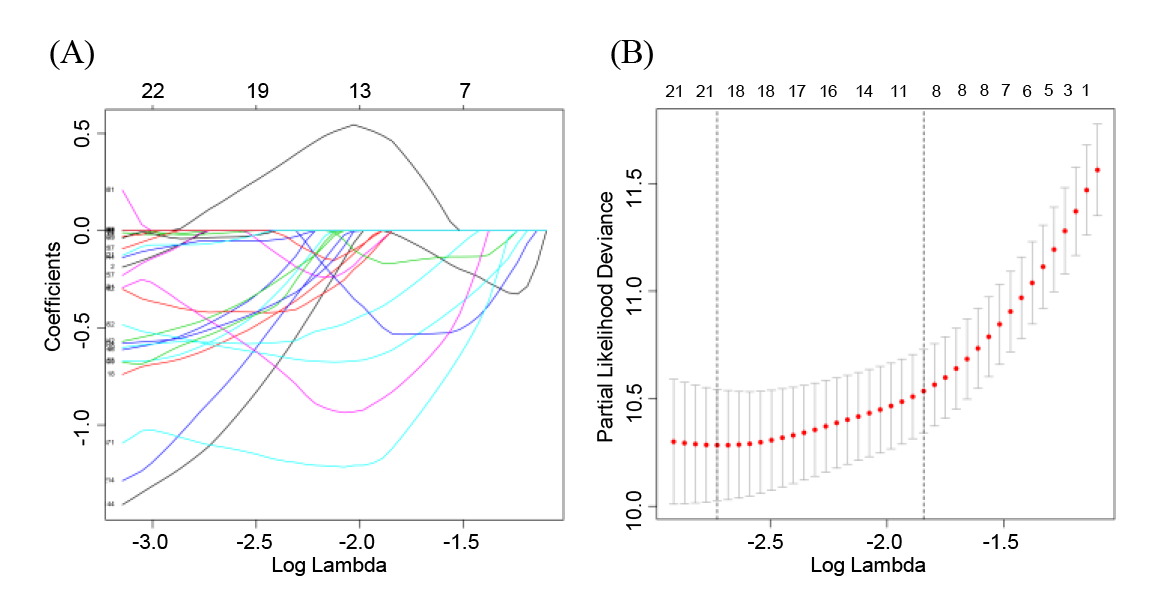

Supplement: Supplementary file 1 [file Image_1.TIF]

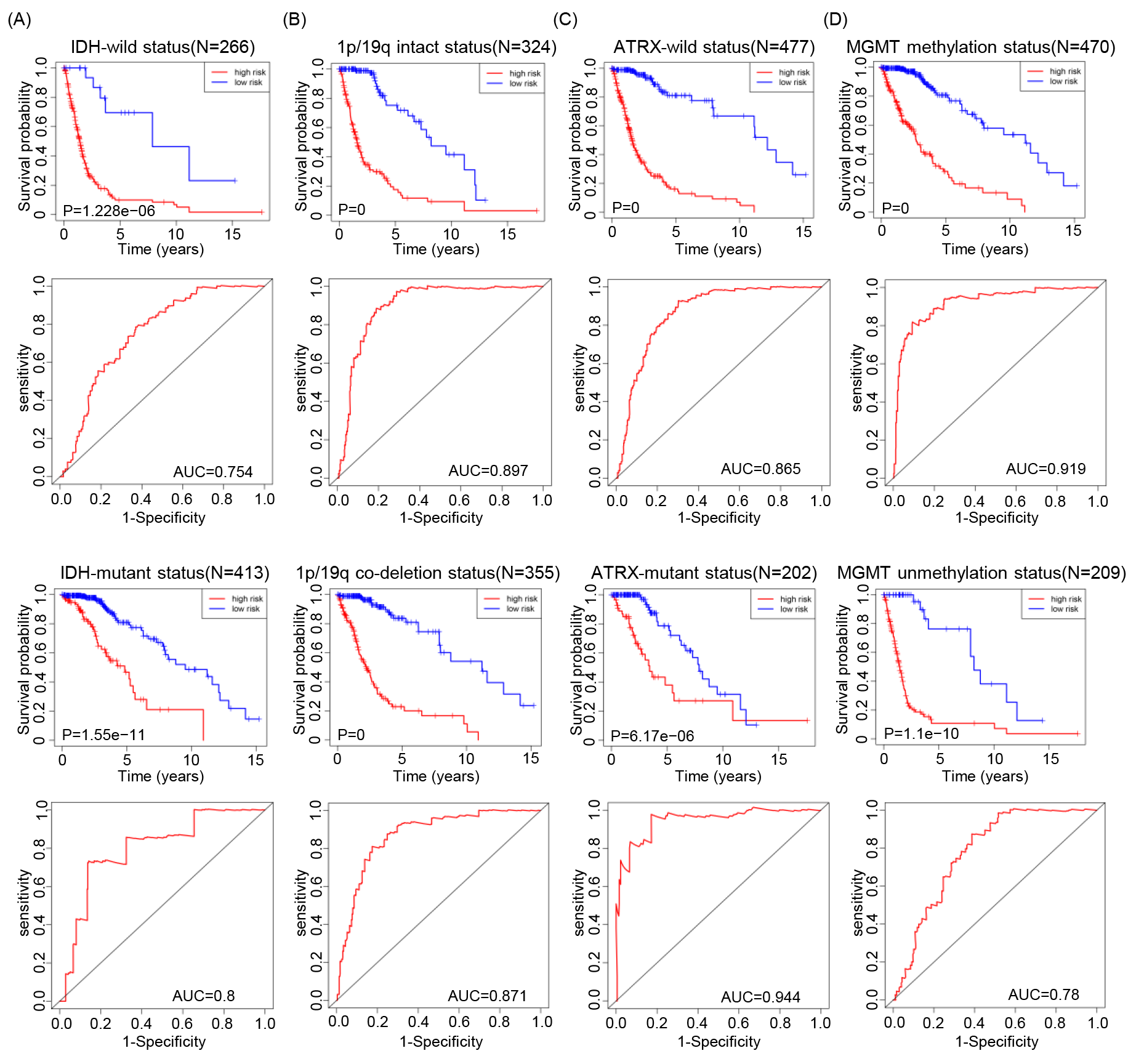

Supplement: Supplementary file 2 [file Image_2.TIF]

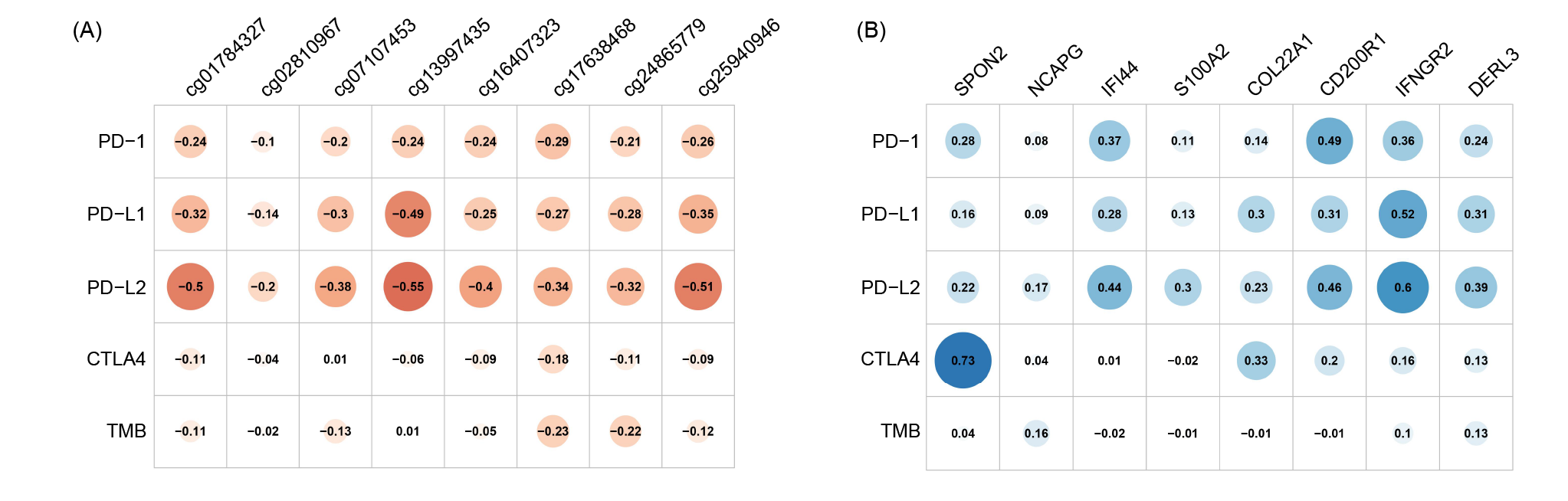

Supplement: Supplementary file 3 [file Image_3.TIF]

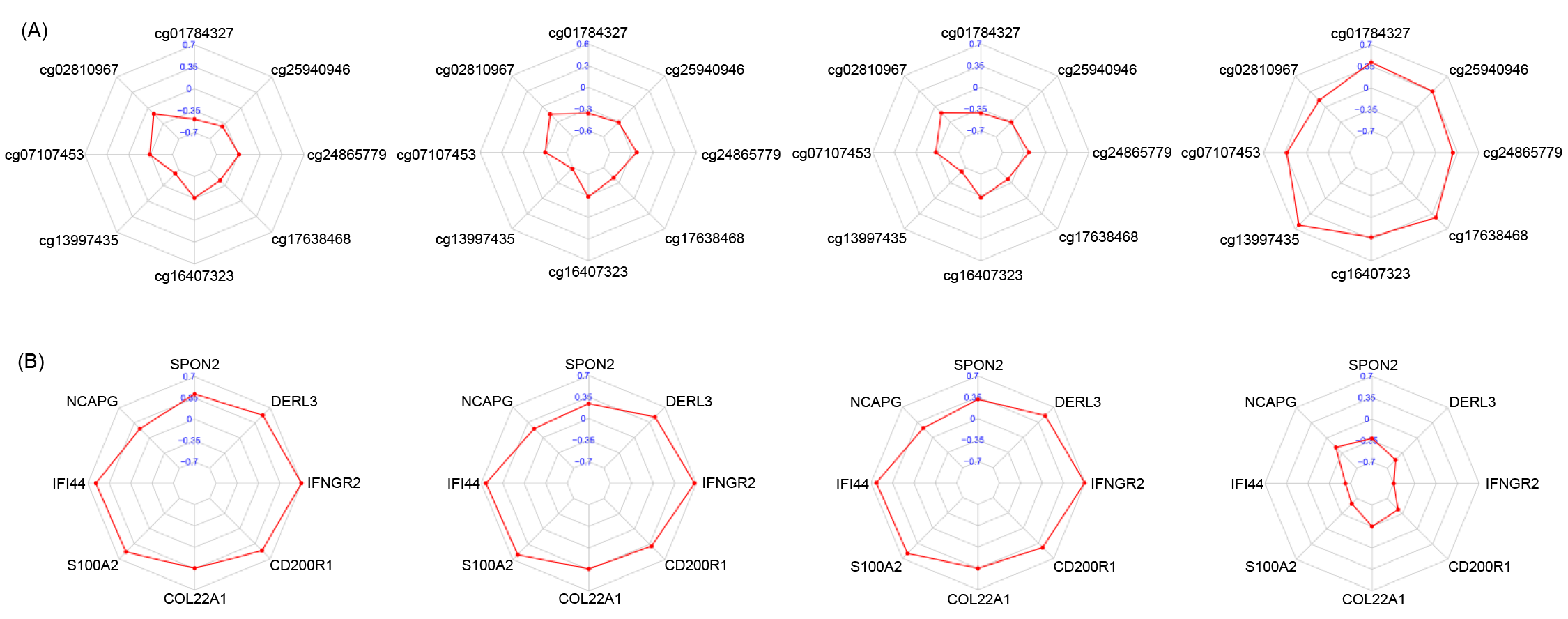

Supplement: Supplementary file 4 [file Image_4.TIF]

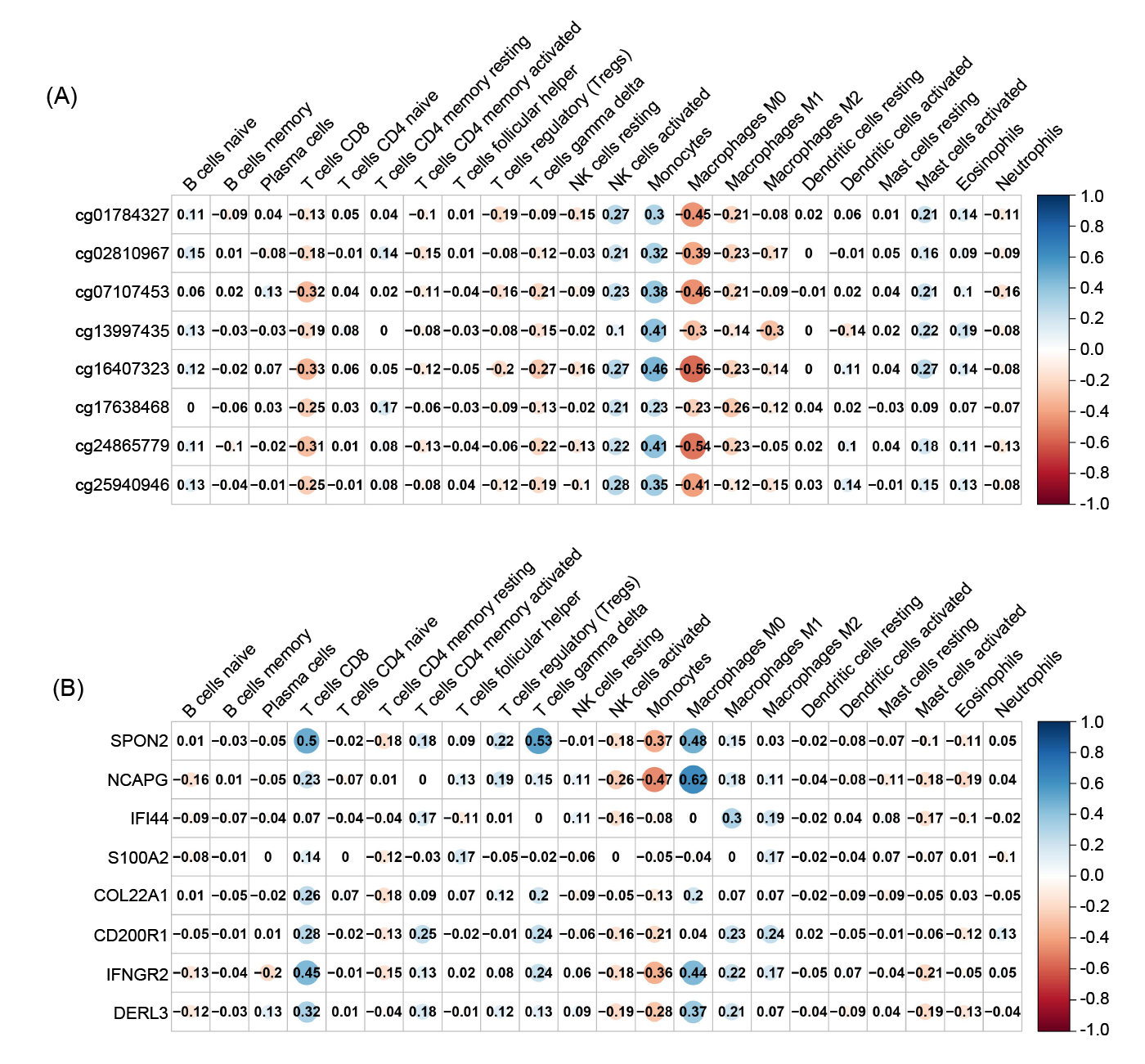

Supplement: Supplementary file 5 [file Image_5.TIF]
