# Supplementary material for: Integrative Analysis of DNA Methylation and Transcriptome Identifies a Predictive Epigenetic Signature Associated With Immune Infiltration in Gliomas
Source: Front Cell Dev Biol. 2021 May 31;9:670854. doi: 10.3389/fcell.2021.670854 (PMC8203203; doi:10.3389/fcell.2021.670854)
Supplement: Supplementary file 6 [file Table_1.DOCX]

Table S1: Clinicopathological characteristics of the glioma patients from TCGA datasets.

| Characteristics | Groups | Entire dataset (n=679) | | Training dataset (n=340) | | Testing dataset (n=339) | | |  |
| --- | --- | --- | --- | --- | --- | --- | --- | --- | --- |
| Age | ＜45 | 332 | (48.9%) | 170 | (50%) | | 162 | (47.8%) | |
|  | 45-59 | 197 | (29%) | 101 | (29.7%) | | 96 | (28.3%) | |
|  | ≥60 | 150 | (22.1%) | 69 | (20.3%) | | 81 | (23.9%) | |
| gender | Female | 302 | (44.5%) | 153 | (45%) | | 149 | (44%) | |
|  | Male | 377 | (55.5%) | 187 | (55%) | | 190 | (56%) | |
| grade | G2 | 258 | (38%) | 133 | (39.1%) | | 125 | (36.9%) | |
|  | G3 | 271 | (39.9%) | 146 | (42.9%) | | 125 | (36.9%) | |
|  | G4 | 150 | (22.1%) | 61 | (18%) | | 89 | (26.2%) | |
| Type | LGG | 529 | (77.9%) | 279 | (82.1%) | | 250 | (73.7%) | |
|  | GBM | 150 | (22.1%) | 61 | (17.9%) | | 89 | (26.3%) | |
| IDH status | Wild | 266 | (39.2%) | 120 | (35.3%) | | 146 | (43.1%) | |
|  | mutant | 413 | (60.8%) | 220 | (64.7%) | | 193 | (56.9%) | |
| ATRX status | Wild | 477 | (70.3%) | 234 | (68.8%) | | 243 | (71.7%) | |
|  | mutant | 202 | (29.7%) | 106 | (31.2%) | | 96 | (28.3%) | |
| 1p19q status | Intact | 324 | (47.7%) | 158 | (46.5%) | | 166 | (49%) | |
|  | Co-deletion | 355 | (52.3%) | 182 | (53.5%) | | 173 | (51%) | |
| the methylation status of MGMT promoter | Methylation | 470 | (69.2%) | 248 | (72.9%) | | 222 | (65.5%) | |
|  | unmethylation | 209 | (30.8%) | 92 | (27.1%) | | 117 | (34.5%) | |
| Radiation therapy | Yes | 403 | (59.4%) | 188 | (55.3%) | | 215 | (63.4%) | |
|  | No | 191 | (28.1%) | 104 | (30.6%) | | 87 | (25.7%) | |
|  | NA | 85 | (12.5%) | 48 | (14.1%) | | 37 | (10.9%) | |
| State | Alive | 436 | (64.2%) | 224 | (65.9%) | | 212 | (62.5%) | |
|  | Dead | 243 | (35.8%) | 116 | (34.1%) | | 127 | (37.5%) | |
