# Supplementary material for: Integrative Analysis of DNA Methylation and Transcriptome Identifies a Predictive Epigenetic Signature Associated With Immune Infiltration in Gliomas
Source: Front Cell Dev Biol. 2021 May 31;9:670854. doi: 10.3389/fcell.2021.670854 (PMC8203203; doi:10.3389/fcell.2021.670854)
Supplement: Supplementary file 7 [file Table_2.DOCX]

Table S2: The percentage of high-and low-risk gliomas in different subtypes from the TCGA database.

|  | Subtypes | Total number | Low risk | High risk |
| --- | --- | --- | --- | --- |
| LGG(n=529) | IDH-wild | 124(23.4%) | 19(15.3%) | 105(84.7%) |
|  | IDH-mutant | 405(76.6%) | 330(81.5%) | 75(18.5%) |
|  | ATRX-wild | 339(64.1%) | 187(55.2%) | 152(44.8%) |
|  | ATRX-mutant | 190(35.9%) | 162(85.3%) | 28(14.7%) |
|  | 1p/19q_intact | 236(44.6%) | 140(59.3%) | 96(40.7%) |
|  | 1p/19q_co-deletion | 293(55.4%) | 209(71.3%) | 84(28.7%) |
|  | MGMT_unmethylation | 121(22.9%) | 44(36.4%) | 77(63.6%) |
|  | MGMT_methylation | 408(77.1%) | 305(74.8%) | 103(25.2%) |
| GBM(n=150) | Classical | 25(16.7%) | 0(0%) | 25(100%) |
|  | G-CIMP | 3(0.4%) | 1(33.3%) | 2(66.7%) |
|  | Mesenchymal | 23(3.4%) | 0(0%) | 23(100%) |
|  | Neural | 10(1.5%) | 0(0%) | 10(100%) |
|  | Proneural | 13(1.9%) | 0(0%) | 13(100%) |
|  | NA | 74(10.9%) | 1(1.4%) | 73(98.6%) |
