# Supplementary material for: Integrative Analysis of DNA Methylation and Transcriptome Identifies a Predictive Epigenetic Signature Associated With Immune Infiltration in Gliomas
Source: Front Cell Dev Biol. 2021 May 31;9:670854. doi: 10.3389/fcell.2021.670854 (PMC8203203; doi:10.3389/fcell.2021.670854)
Supplement: Supplementary file 8 [file Table_3.DOCX]

Table S3: Association the epigenetic signature with clinical and molecular features in gliomas from the TCGA database.

|  | Subtypes | No.(%) | Low risk  No.(%) | High risk  No.(%) | Chi-square test  P values |
| --- | --- | --- | --- | --- | --- |
| age | <45 | 332(48.9%) | 240(72.3%) | 92(27.7%) | <0.0001 |
|  | 45-59 | 197(29%) | 86(43.7%) | 111(56.3%) |  |
|  | >=60 | 150(22.1%) | 25(16.7%) | 125(83.3%) |  |
| gender | male | 377(55.5%) | 200(53.1%) | 177(46.9%) | 0.4293 |
|  | female | 302(44.5%) | 151(50%) | 151(50%) |  |
| grade | G2 | 258(38%) | 206(79.8%) | 52(20.2%) | <0.0001 |
|  | G3 | 271(39.9%) | 143(52.8%) | 128(47.2%) |  |
|  | G4 | 150(22.1%) | 2(1.3%) | 148(98.7%) |  |
| IDH status | wild | 266(39.2%) | 19(7.1%) | 247(92.9%) | <0.0001 |
|  | mutant | 413(60.8%) | 332(80.4%) | 81(19.6%) |  |
| ATRX status | wild | 477(70.3%) | 188(39.4%) | 289(60.6%) | <0.0001 |
|  | mutant | 202(29.7%) | 163(80.7%) | 39(19.3%) |  |
| MGMT methylation status | methylated | 470(69.2%) | 307(65.3%) | 163(34.7%) | <0.0001 |
|  | unmethylated | 209(30.8%) | 44(21.1%) | 165(78.9%) |  |
| 1p/19q status | intact | 324(47.7%) | 140(43.2%) | 184(56.8%) | <0.0001 |
|  | co-deletion | 355(52.3%) | 211(59.4%) | 144(40.6%) |  |
